# Self-reported preparedness of final year undergraduate dental students and interns in Saudi Arabia: a multi-institutional study

**DOI:** 10.1186/s12909-024-05246-z

**Published:** 2024-03-13

**Authors:** Muhammad Qasim Javed, Ayman Moaz Abulhamael, Zaina Ahmad, Muhammad Muhammad, Muhammad Ali Faridi, Kiran Imtiaz Khan, Syed Rashid Habib, Kamran Ali

**Affiliations:** 1 Department of Conservative Dental Sciences, College of Dentistry, Qassim University, 51452 Buraidah, Qassim, PO Box 1162, Saudi Arabia; 2 Department of Endodontics, Faculty of Dentistry, King Abdulaziz University, 21589 Jeddah, P.O. Box 80209, Saudi Arabia; 3 Department of Operative Dentistry, Islamic International Dental College and Hospital, Riphah International University, Islamabad, Pakistan; 4 Restorative Dental Sciences Department, College of Dentistry, Imam Abdulrahman bin Faisal University, Dammam, Saudi Arabia; 5 Department of Operative Dentistry, Frontier Medical and Dental College, Abbottabad, KPK Pakistan; 6 Department of Prosthetic Dental Sciences, College of Dentistry, King Saud University, King Abdullah Road, 11545 Riyadh, P. O. Box 60169, Saudi Arabia; 7 College of Dental Medicine, Qatar University, 2713 Doha, Qatar

**Keywords:** Clinical competence, Dental, Education, Self-report

## Abstract

**Background:**

Contemporary undergraduate dental education aims to equip the dental students with clinical competence, empathy, and professionalism to enable them to deliver safe and effective dental care to the communities. The purpose of this study was to assess the self-reported preparedness of final year dental students and interns at three Saudi dental institutions, using the pre-validated Dental Undergraduates Preparedness Assessment Scale (DU-PAS).

**Methods:**

A cross-sectional study design was employed to assess the self-reported preparedness of the participants using the DU-PAS. Following ethics approval, a probability sampling technique was used to recruit undergraduate dental students and interns from three dental institutions in Saudi Arabia. The data was collected online on Google Forms and all participants provided their consent to participate in the study prior to providing their responses to DU-PAS.

**Results:**

Responses were received from 397 participants including 171 males and 226 females yielding a response rate of 60.3%. The total mean score of the participants was 81.85 ± 13.11. Although higher scores were reported in males, the interaction between gender and DU-PAS scores were not significant. Interaction between DU-PAS scores and stage of education showed significant effect of the stage of education with interns reporting higher overall scores. The participants reported that they were able to perform most clinical procedures independently. However, low confidence was reported in performing multi-rooted endodontics, fabrication of removable dentures and orthodontic assessment. The participants also expressed their confidence in a wide range of behavioural attributes related to communication, teamworking and professionalism. However, lack of experience was noted in referral for oral cancer, interpreting research, and evaluation of new dental products using an evidence-based approach.

**Conclusion:**

The study provides useful insights into the self-reported preparedness of undergraduate dental students and interns in three dental institutions. While the overall preparedness of the participants was comparable to their international peers, the findings underscore the need for further enhancements to the teaching and training of undergraduate students particularly in multirooted endodontics, removable prosthodontics and orthodontics.

## Introduction

The landscape of contemporary healthcare education is shaped by societal needs and demands for safe and effective clinical services. All dental professionals are expected to demonstrate competence in clinical practice, empathy, and professionalism to enable them to deliver safe and effective clinical care [1, 2]. Regulators of healthcare education expect the education providers to ensure that the students gain clinical competence and experience prior to graduation [3]. The standards of clinical education emphasize the acquisition of scientific knowledge, development of psychomotor skills in simulated and clinical settings under trained and experienced supervisors, and the use of valid and reliable methods of assessment [4].

Undergraduate dental education requires undergraduate students to develop competence in a wide range of invasive and irreversible clinical procedures on real patients under supervision of qualified dental professionals [5]. Dental treatment warrants a fine balance between functionality and aesthetics to ensure the predictability and longevity of the treatment whilst meeting the patients’ expectations [6]. Dental professionals not only provide management of oral diseases but also contribute to enhancing patients’ self-esteem and appearance. Moreover, dental treatment usually requires invasive interventional procedures carried out within a confined space, demanding high standards of manual dexterity and precision [7]. Alongside technical expertise, dental professionals are expected to display effective communication skills to manage stress associated with dental treatment, time management, teamwork, leadership, and high standards of professionalism [8].

Dental graduates face a multitude of challenges in order to prepare themselves for a smooth transition from a dental school into independent clinical practice. Preparedness of dental graduates is an overarching concept and encompasses a wide range of attributes related to cognition, and development of clinical and affective skills [9]. Preparedness of dental graduates is a topic of fundamental significance in dental education. Research in undergraduate dental education involving pedagogy, clinical training, curriculum development and assessment methods is ultimately aimed at informing the stakeholders how to prepare the dental graduates for their future careers [10, 11]. 

Existing literature shows that whilst most dental students achieve competence in basic procedures, some skills such as comprehensive treatment planning, multi-rooted endodontics, surgical tooth extractions, and complex restorative treatment are more difficult to master [8, 10, 12–14]. Therefore, it is imperative for dental educators to evaluate the preparedness of students in dentistry throughout their clinical training, so the gaps are identified early, and appropriate remediation is provided to underperforming students.

The aim of the study was to evaluate self-reported preparedness of final-year dental students and interns at three public universities in Saudi Arabia using the pre-validated Dental Undergraduates Preparedness Assessment Scale (DU-PAS) [15]. 

## Methods

### Ethics approval

Ethical approval for this study was obtained from the institution research ethics committee, Qassim University Saudi Arabia (Approval number 21-16-07). Participation in the study was voluntary. All data was collected and processed anonymously and individual participants in the study are not identifiable. The research data was stored securely and was accessible to the research team only.

### Research question

How confident are final year dental undergraduate students and interns in Saudi Arabia about their readiness for independent clinical practice?

### Study design

This study was carried out using an analytical cross-sectional research design.

### Settings

It was a multi-institutional study carried out at three public universities in Saudi Arabia namely, Qassim University, Buraydah, King Abdulaziz University, Jeddah, and Imam Abdul Rahman bin Faisal University, Dammam.

### Participants and sampling technique

The target participants for this study were final-year undergraduate dental students (*n* = 331) and interns(*n* = 328). A probability sampling technique was used to recruit the participants.

### Sample size calculation

To ensure a representative sample, a purposive sampling technique was employed. An acceptable sample size of 331 was identified based on a confidence level of 99%, a margin of error of 5%, and a total population size of 659, using a Rao soft Sample Size Calculator [16]. 

### Research instrument

This study utilized the pre-validated Dental Undergraduates Preparedness Assessment Scale (DU-PAS) [15] This instrument has been developed using item-response theory (IRT) psychometric model and has been widely used in multiple studies [14, 17, 18]. It consists of 50 items divided in two sections. Section A has 24 items related to clinical skills and responses are measured on a three -point scale: 0 = no experience; 1 = with help; and 2 = independently. Section B has 26 items related to cognitive and behavioural attributes and is also measured on a three-point scale: 0 = no experience, 1 = mostly and 2 = always. The cumulative score on DU-PAS is100.

### Data collection

Data were collected online, with invitations sent to the potential participants’ official emails and official WhatsApp groups. Participants were provided with an information sheet explaining the purpose and scope of the study and were asked to provide informed consent before providing responses to the questionnaire. Participants were invited to provide responses to DU-PAS questionnaire online using Google Forms during a four-week window. A reminder was sent at the end of the third week.

### Data analysis

Data was analysed using SPSS version 24 (IBM Corp,32 Armonk, N.Y., US). Descriptive statistics were calculated to describe the sample and subgroups, and their distributions of scores for each part, and between genders. Chi-squared tests of association were conducted to compare the distribution of response options between groups where they were treated categorically, with independent *t*-tests were used where responses were treated as numeric. A *p* value < 0.05 was considered to be significant.

## Results

Responses were received from 397 participants including 248 students and 149 interns, yielding a response rate of 60.3%. The response rate for students was 74.9%, while interns’ response rate was 45.4%. The demographic characteristics of the participants are summarized in Table 1. The age range of participants was 22 to 29 years (mean age = 24.04 years ± 1.40 and included 171 males, and 226 females.

**Table 1:** Demographic Breakdown profile of the participants

Characteristic		Frequency (N)	Percentage (%)	Mean age (Years) ± SD
Age				24.04 (1.40)
Gender				
	Male	171	43.0	24.18 (1.06)
	Female	226	56.8	23.94 (1.60)
Stage of education				
	Final year students	248	62.3	23.66 (1.49)
	Dental interns	149	37.4	24.66 (0.97)

The distribution of participants’ scores on items in Part A of DU-PAS are summarized in Table 2. Self-reported preparedness was high in several clinical skills including prescribing radiographs, comprehensive treatment planning, and effective removal of caries. Confidence was also high in administering local anaesthetic injections, non-surgical periodontal treatment, single-rooted endodontics, crown preparations and non-surgical tooth extractions. A substantial number of respondents reported low scores in some clinical skills. Specifically, 24.9% reported lack of experience in amalgam fillings, and 13.1% in carrying out endodontics of multi-rooted teeth. Participants’ scores were also low in assessment of orthodontic treatment needs of patients.

**Table 2:** Participant scores on Part A of DU-PAS by item

Item	Question	No Experience (%)	With Help (%)	Independently (%)
A1	I am able to obtain a complete medical history from my patients.	1.3	5.8	92.7
A2	I am able to undertake a comprehensive, clinical oral examination	1.5	12.1	86.2
A3	I am able to prescribe appropriate dental radiographs	0.8	7.3	91.7
A4	I am able to undertake periapical radiographs	1.8	11.1	86.9
A5	I am able to undertake bitewing radiographs	1.3	10.8	87.7
*A6	I am able to interpret common findings on dental radiographs	1.0	14.8	83.9
A7	I am able to assess the treatment needs of patients requiring orthodontics	6.3	45.2	48.2
A8	I am able to formulate a comprehensive treatment plan which addresses all treatment needs of my patients	0.8	31.7	67.3
A9	I am able to provide a range of treatment options to my patients based on their individual circumstances	1.0	26.1	72.6
A10	I am able to explain the merits and demerits of various treatment options to my patients	1.0	24.4	74.4
A11	I am able to obtain a valid consent from my patients prior to undertaking any treatment.	2.0	10.3	87.4
A12	I am able to carry out patients’ treatment sessions in an appropriate order	1.5	16.8	81.4
*A13	I am able to prescribe drugs to my patients appropriately	3.3	41.5	55.0
A14	I am able to administer inferior dental nerve blocks effectively	2.0	10.1	87.7
A15	I am able to perform non-surgical periodontal treatment using appropriate methods	5.3	10.3	84.2
A16	I am able to remove dental caries effectively	0.5	8.0	91.2
A17	I am able to restore teeth with tooth-coloured fillings appropriately	1.5	11.1	87.2
A18	I am able to restore teeth with amalgam fillings appropriately	24.9	28.1	46.7
A19	I am able to perform endodontic treatment on single rooted teeth appropriately	2.0	7.8	89.9
A20	I am able to perform endodontic treatment on multi rooted teeth appropriately	13.1	45.0	41.7
A21	I am able to provide crowns using principles of tooth preservation	1.8	23.9	74.1
A22	I am able to provide mechanically sound cast partial dentures	5.8	53.0	41.0
A23	I am able to provide mechanically sound/safe and functioning full dentures	5.0	53.0	41.7
A24	I am able to undertake non-surgical tooth extractions appropriately	1.8	19.1	78.9

The distribution of participants’ scores on items in Part B of the questionnaire is summarized in Table 3. It can be seen that the majority of the participants reported adequate skills related to communication, and teamworking; recognised their limitations in clinical practice; and were able to maintain accurate clinical records. However, 17.3% of participants indicated that they had no experience in the use of evidence-based approaches for assessing new materials and products. Additionally, 14.6% of the respondents lacked confidence in referring suspected oral cancer patients.

**Table 3:** Participant scores on Part B of DU-PAS by item

Item	Question	No Experience (%)	Mostly (%)	Always (%)
B25	I feel I can manage peoples’ expectations of their treatment	1.5	54.8	43.5
B26	I feel able to motivate my patients to encourage self-care for their dental needs	1.3	39.7	58.8
B27	I recognise my personal limitations in clinical practice	0.8	25.4	73.6
*B28	I feel comfortable asking for help from supervisor or colleague if needed	1.5	26.6	71.6
B29	I am able to refer patients with complex treatment needs appropriately	0.8	24.6	74.4
B30	I feel confident referring patients with suspected oral cancer	14.6	29.4	55.8
B31	I reflect on my clinical practice in order to address my learning needs	1.3	38.9	59.5
B32	I have sufficient knowledge of scientific principles which underpin/support my dental practice	1.8	45.5	52.5
B33	I am confident to evaluate new dental materials and products using an evidence-based approach	17.3	47.5	34.9
B34	I am confident to interpret the results of research which may influence my practice	10.8	50.3	38.7
B35	I use an evidence-informed approach in my clinical practice.	10.6	43.2	46.0
B36	I feel I can manage to communicate effectively with my patients	1.5	26.6	71.6
B37	I provide opportunities for my patients to express their expectations from dental treatment	1.5	29.4	68.8
B38	I feel confident to address barriers for effective communication with patients appropriately	1.5	32.7	65.6
B39	I feel confident to communicate potential risks of operative procedures to patients	1.5	26.1	72.1
B40	I feel confident to communicate appropriately with my colleagues	1.8	21.1	76.9
B41	I feel confident managing anxious patients with appropriate behavioural techniques	5.5	40.7	53.5
B42	I am able to manage the behaviour of children to enable appropriate dental treatment	9.5	47.2	43.0
B43	I am able to fulfil my responsibilities as an effective member of the dental team	1.8	29.6	68.3
B44	I maintain accurate records of my clinical notes	2.8	30.9	66.1
B45	I am able to work within the constraints of clinical appointment schedules	2.5	39.9	57.3
B46	I take responsibility for my continuing professional development	1.0	30.4	68.3
B47	I am aware of my legal responsibilities as a dental professional	2.3	29.9	67.6
B48	I restrict my relations with my patients to a professional level	1.0	32.2	66.6
*B49	I feel able to raise concerns about inappropriate behaviour of my colleagues	6.5	34.4	58.8
B50	I take appropriate measures to protect patient confidentiality	1.0	24.9	73.9

The total mean score of the participants was 81.85 ± 13.11. Interaction between gender and DU-PAS scores showed that the scores of males were higher overall, as well as for Part A and Part B of DU-PAS as shown in Table 4. However these gender-related differences were statistically not significant.

**Table 4:** Descriptive statistics for DU-PAS scores by gender

Part	Gender	*n*	Mean	SD	Min	Max	Range	IQR	*p*-value
Overall	Male	171	82.61	13.04	43.00	100.00	57.00	18.00	
	Female	226	81.27	13.16	26.00	100.00	74.00	18.00	
	Total	397	81.85	13.11	26.00	100.00	74.00	18.50	0.996
Part A (24 items)	Male	171	41.31	5.87	21.00	48.00	27.00	6.00	
	Female	226	40.72	6.39	0.00	48.00	48.00	7.00	
	Total	397	40.97	6.17	0.00	48.00	48.00	7.00	0.826
Part B (26 items)	Male	171	41.30	8.68	20.00	52.00	32.00	14.00	
	Female	226	40.55	8.49	12.00	52.00	40.00	13.00	
	Total	397	40.88	8.57	12.00	52.00	40.00	13.00	0.427

Interaction between DU-PAS scores and stage of education showed significant effect of the stage of education as shown Table 5. Compared to the final year students, the interns reported higher overall scores and for Part A of DU-PAS (*p*<-.05). Although the scores of interns were higher for Part B as well, these differences were not statistically significant. **Discussion**.

**Table 5:** Descriptive statistics for DU-PAS scores by stage of education

Part	Stage	*n*	Mean	SD	Min	Max	Range	IQR	*p*-value
Overall	Final year students	248	80.63	12.13	35.00	100.00	65.00	17.00	
	Dental interns	149	83.87	14.42	26.00	100.00	74.00	20.00	
	Total	397	81.85	13.11	26.00	100.00	74.00	18.50	**0.040**
Part A (24 items)	Final year students	248	40.58	5.28	22.00	48.00	26.00	6.00	
	Dental interns	149	41.63	7.40	0.00	48.00	48.00	7.00	
	Total	397	40.97	6.17	0.00	48.00	48.00	7.00	**0.001**
Part B (26 items)	Final year students	248	40.06	8.39	12.00	52.00	40.00	13.00	
	Dental interns	149	42.24	8.71	20.00	52.00	32.00	12.50	
	Total	397	40.88	8.57	12.00	52.00	40.00	13.00	0.072

*p values < 0.05 were significant and are highlighted in bold

To our knowledge, this is the first multi-institutional study to evaluate the preparedness of both Saudi dental students and interns using DU-PAS. This article explores preparedness of the participants for independent clinical practice and identifies their strengths, weaknesses and along with recommendations for future pedagogical improvements in undergraduate dental curricula in Saudi Arabia. The findings of this study suggest that that the participants showed an adequate level of preparedness overall as indicated by a mean score of 81.85 ± 13.11. The mean score is higher than reported in previous studies utilizing DU-PAS in Malaysia (79.56), Pakistan (65.60), and the United Kingdom (74.00), suggesting that the participants in this study reported a better level of preparedness compared to their peers in the aforementioned countries [20–22]. The dental interns reported significantly higher scores overall and for Part A items related to clinical skills compared to final year students. These findings are consistent with increased clinical experience gained during the internship year.

Regarding weaknesses in clinical skills, over 50% of participants needed assistance in the fabrication of removable prosthesis, which could be attributed to the technical complexity of multiple steps involved in making dentures. Dental educators need to identify the specific gaps in the skill set related to removable prosthodontics and address these by providing further opportunities for consolidation. Moreover, students may be given opportunities to shadow experienced prosthodontists in clinical settings to improve their confidence.

Endodontics on multi-rooted teeth was another area of weakness with just over 50% participants expressed lack of experience and/or the need for assistance from their supervisors. Multi-rooted endodontics has been widely reported to be challenging for dental students and new graduates in multiple studies [14, 17, 19, 20]. The lack of confidence is related to a combination of technical difficulties in performing endodontics on multi-rooted teeth as well as limited availability of suitable patients [21]. Dental educators may address this by providing additional consolidation sessions in simulated settings allowing students to practice on artificial and natural teeth [22]. Structured remediation with close supervision and individualised feedback to underperforming students may be used to improve their confidence in multi-rooted endodontics.

The third key area of weakness reported by the participants was related to their ability to assess orthodontic treatment needs of patients. Low confidence in assessing orthodontic treatment needs of patients amongst undergraduate students is widely reported in the literature and a recent scoping review has heighted significant variations in the scope of undergraduate curricula in orthodontics globally [23]. Given the remit of general dentists is primarily related to recognition of orthodontic problems and referral to specialists, assessment of orthodontic needs of patients needs to be the core focus of undergraduate dental curricula in orthodontics [2, 24]. 

A large percentage of the participants reported a lack of experience in referral of suspected oral cancer patients. This lack of confidence may be attributed to a lack of clinical exposure to patients with oral cancer cases in comparison to routine dental problems. It is a well-recognized gap in dental education that can be bridged through structured clinical exposure to specialist settings in oral and maxillofacial surgery, use of case-based discussions and clinical problem-solving sessions [25]. 

Only a small fraction of the respondents expressed confidence in evaluating new dental materials and products using an evidence-based approach. These findings corroborate with other published studies which report limited confidence in research methodology and evidence-based practice amongst undergraduate dental students [26, 27]. Historically, undergraduate students have not always had adequate exposure to research methodology and have had limited opportunities to conduct research [28]. There is a growing recognition of the gaps in the research skills of undergraduate dental students, and many dental institutions now require students to develop skills in conducting critical appraisal of literature and undertake research projects as part of their undergraduate curriculum [27, 29–31]. 

The lack of experience in managing pediatric patients is a common concern among dental students and interns globally [32]. This is also reflected in the present study where less than 50% of the participants reported confidence in managing children in order to deliver effective treatment. Pediatric dentistry demands a unique skill set and approach due to the distinct emotional and psychological needs of children. Limited exposure to pediatric patients during education and training is likely to impact adversely on the confidence of dental students and interns. Issues such as dental anxiety, uncooperative behaviour, and the need for effective communication with both children and their parents can further add to the difficulties associated with managing paediatric patients [32, 33]. Student placements with specialists in pediatric dentistry can provide opportunities to shadow experienced clinicians and improve their confidence in managing children and expectations of their parents.

Given the findings of this study, it is recommended that the participating institutions need to revisit undergraduate teaching in removable prosthodontics, and endodontics on multi-rooted teeth. Simulated laboratory settings can be used to provide opportunities for consolidating these skills in a non-threatening environment followed by appropriate experience on patients in clinical settings [34]. Weaknesses related to orthodontic assessment and oral cancer referral may be addressed by utilizing contemporary strategies such as case-based learning (CBL) which provide opportunities with active student participation [35–37]. In addition, clinical placements of students in orthodontics and maxillofacial surgery are suggested to allow students exposure to assessment of patients by relevant specialists to enhance their confidence. Finally dental educators in Saudi Arabia may consider boosting student learning in critical appraisal of literature to equip them with the knowledge and skills to evaluate evidence supporting the use of new materials, and treatments.

The main limitation of this study is that the findings are based on self-reported confidence of the participants, and it is possible that the scores may be inflated. Nevertheless, the participants also acknowledged weaknesses in several attributes highlighting the need for improvements in undergraduate teaching at the participating institutions.

In summary, our study sheds light on the readiness of dental students and interns in Saudi Arabia and identifies some deficiencies in dental education that can leave students ill-prepared to confront the challenges encountered during their clinical training years. In its pursuit, the study aligns with the global discussion that highlights the importance of aligning dental education with existing healthcare needs to ensure a seamless transition for graduates into their professional dental practice Additionally, it sets the stage for future research to compare these findings with data from other dental institutions.

## Conclusion

This study provides insights into the strengths and weaknesses in the preparedness of undergraduate dental students and interns. Although the participants reported confidence and proficiency in several clinical skills, improvements are warranted to enhance skills related to endodontics on multirooted teeth, provision of removable prosthesis, and orthodontic assessment Regarding behavioural attributes, the participants expressed confidence in their communication skills, but low confidence was reported in managing patient expectations, oral cancer referral, and evaluating evidence related to new materials and products. These findings underscore the need for improvements in the teaching and learning of dental students in the participating institutions to address gaps in their clinical skills and behavioural attributes.

## Data Availability

Data will be made available by the corresponding author upon reasonable request.
